# Can Impacts of Climate Change and Agricultural Adaptation Strategies Be Accurately Quantified if Crop Models Are Annually Re-Initialized?

**DOI:** 10.1371/journal.pone.0127333

**Published:** 2015-06-04

**Authors:** Bruno Basso, David W. Hyndman, Anthony D. Kendall, Peter R. Grace, G. Philip Robertson

**Affiliations:** 1 Department of Geological Sciences, Michigan State University, East Lansing, MI, United States of America; 2 W. K. Kellogg Biological Station, Michigan State University, Hickory Corners, MI, United States of America; 3 Department of Plant, Soil, and Microbial Sciences, Michigan State University, East Lansing, MI, United States of America; 4 Institute of Future Environments, Queensland University of Technology, Brisbane, Australia; Ghent University, BELGIUM

## Abstract

Estimates of climate change impacts on global food production are generally based on statistical or process-based models. Process-based models can provide robust predictions of agricultural yield responses to changing climate and management. However, applications of these models often suffer from bias due to the common practice of re-initializing soil conditions to the same state for each year of the forecast period. If simulations neglect to include year-to-year changes in initial soil conditions and water content related to agronomic management, adaptation and mitigation strategies designed to maintain stable yields under climate change cannot be properly evaluated. We apply a process-based crop system model that avoids re-initialization bias to demonstrate the importance of simulating both year-to-year and cumulative changes in pre-season soil carbon, nutrient, and water availability. Results are contrasted with simulations using annual re-initialization, and differences are striking. We then demonstrate the potential for the most likely adaptation strategy to offset climate change impacts on yields using continuous simulations through the end of the 21^st^ century. Simulations that annually re-initialize pre-season soil carbon and water contents introduce an inappropriate yield bias that obscures the potential for agricultural management to ameliorate the deleterious effects of rising temperatures and greater rainfall variability.

## Introduction

Sustainable food production under a changing climate is among the most important international research priorities [1,2]. There is a global consensus among climate and agricultural scientists about the need to quantify the likely impacts of climate change on crop yields due to their significant consequences on both food prices and the global economy [3–5].

Estimates of climate change impacts on global food production are usually based on statistical and process-based simulation models [1,6–8]. Statistical models have been useful to assess the impacts of historic trends on yields [9]. However, they are not well suited to estimate climate change impacts on future yields because they cannot capture: 1) changes in soil carbon and biophysical properties due to management practices (e.g., effects of long-term no tillage practices and retention of crop residues on the soil surface), 2) the impacts of long term increases in temperature (which is projected to change well outside the historically observed range) on yields, soil carbon, and water, as well as 3) the influence of increasing atmospheric CO_2_ concentrations (which are similarly beyond the range of historical data) on plant growth. Process-based agroecosystem models, on the other hand, simulate crop yields as a function of climate, CO_2_ concentrations, soil properties (soil organic matter content, water holding capacity, and nitrogen availability), crop genetics (cultivars, growth and development rates), and agronomic management (tillage, planting date, fertilization, irrigation) [10–14]. For these reasons, process-based models are predictive and tend to be more appropriate than statistical models when predicting future crop yields under a changing climate as they can account for the impact of changes in climate, soil, management and cultivars on crop yield [3,7,8,15,16].

Most commonly, applications of process-based models to project future yields under climate change re-initialize soil water, carbon, and nutrient conditions to the same state each year. If the purpose of the simulation is solely to assess the impact of changes in weather independently from other variables on yield, then such an approach may be justifiable—though it still cannot account for non-linear interactions. Such an approach should not be used to identify adaptation and mitigation strategies because soils will change through time as a consequence of management practices. The vast majority of published studies that examine climate change impacts on crop production have used this approach [7; 17].

Reinitializing a model specifies that soil conditions are the same each year at or near the beginning of the growing season. This is a reasonable assumption for static soil properties such as texture, but it is not reasonable for dynamic properties such as soil moisture, carbon, and nutrient (nitrogen, sulfur, and phosphorus) levels, many of which are critical to accurately simulate both short- and long-term yields. For example, soil water content at a given initialization time can dramatically differ if fallow period rain and snowmelt fails to completely recharge the soil profile, which is often the case in non-humid environments. Soil organic matter can change substantially over decades in response to management [18] and climate [19] triggering concomitant changes in soil water, nitrogen and other nutrients. Tillage management can significantly impact root-zone soil quality through mechanical alteration of soil structure including compaction effects on infiltration and drainage. If simulations neglect to include year-to-year changes in initial soil water or changes in soil conditions related to agronomic management, adaptation and mitigation strategies designed to maintain stable yields under climate change cannot be properly evaluated.

Here we demonstrate the importance of simulating crop yields using a continuous model that accounts for annual carryover of soil water, nutrients, and carbon over long time periods, such as in climate change impact and adaptation studies. This allows for a more realistic assessment of management factors, such as tillage practice that might exacerbate or attenuate climate change impacts on yields and yield stability, and thereby provide insights into long-term adaptation strategies. We then use the continuous model to evaluate the most likely adaptation strategy to select different cultivars, shift planting dates, and alter plant densities.

## Methods

### Study Site—High Plains, Nebraska, USA

We examine climate and soils for a representative region that is well suited for maize production in the Northern High Plains (NHP), USA. More specifically, we chose a NHP demonstration site in Cuming County, Nebraska where the regional climate is dry sub-humid with an average annual precipitation from 1981–2010 of 740 mm, of which 495 mm falls during an average growing season (April-September). The average annual temperature is 10°C with a monthly maximum of 24°C in July and a monthly minimum of—6°C in January. The soil texture is silty-clay-loam with an average soil organic carbon content of 1.8%. For the decade from 2003–2012, ~58,000 ha of maize was harvested in Cuming County, of which 24% was irrigated [20,21].

### SALUS Crop Model

We simulated maize yields using the SALUS (System Approach to Land Use Sustainability); [22, 23] model for a 121 year period (1979–2099). SALUS is a process-based model derived from the well-established and validated CERES model that is designed to quantify the impact of management strategies and their interactions with the soil-plant-atmosphere system on yield and carbon (C), Nitrogen (N), and Phosphorous (P) dynamics. The model simulates daily crop growth and soil, water, and nutrient conditions under different management strategies for multiple years [22]. SALUS accommodates various crop rotations, planting dates, plant populations, irrigation, fertilizer applications (organic and inorganic), and tillage practices to simulate daily plant growth and soil processes during both the growing season and fallow periods. Growth is primarily determined by the Radiation Use Efficiency (RUE) approach and is then reduced based on transpiration and nitrogen limitations. The effects of CO_2_ are simulated by adjusting RUE as a function of CO_2_ with corresponding changes in transpiration. The soil water balance module has advanced from the CERES models with improvements in calculations of infiltration, drainage, evapotranspiration, runoff, root growth, and water uptake [13, 24]. SALUS converts snow accumulation into precipitation that is later distributed as infiltrating water. A new cold hardiness routine accounts for the effects of very low temperatures on winter cereals such as wheat. The model simulates SOM and N dynamics from three soil organic carbon pools (active, slow and passive) and two crop residue/fresh organic matter pools (structural and metabolic). The soil P model incorporates inorganic and organic P dynamics, with inorganic P divided into labile, active, and stable pools.

The SALUS model does not explicitly include sub-models to simulate pest and disease outbreaks or the occurrence of extreme weather events such as hail. It has been tested for crop yield (e.g. [23, 25, 26, 27, 28]), soil C dynamics (e.g. [22]), plant N uptake and phenology (e.g. [22, 29]), nitrate leaching (e.g. [30, 31]), as well as tillage effects on soil properties [29, 32, 33]. The initial fraction of three SOC pools in the continuous runs shown in this paper was determined following the procedure described in [33].

Simulations for this study were performed for the 121 year period in both continuous and re-initialization modes. For the continuous mode, the simulations account for changes in soil C, N, and water content during both growing seasons and fallow periods.

In re-initialization mode, soil water, nutrient, and carbon levels were reset each year to the initial values on January 1. In re-initialized simulations, Plant Extractable Soil Water (PESW)—defined as the water content between the drained upper limit and the lower limit for the soil profile)—was reset to the Drained Upper Limit—DUL—of soil water content (35% for this silty clay loam soil). This value is representative for early season water content of the soils in this region, which commonly have adequate water from snowmelt and fall to early spring precipitation, with no crop water use during this period. This is a reasonable assumption as soils drain to this moisture content during a relatively short time period after rain or snowmelt. Soil nitrogen levels were reset to 20 kg N/ha at planting and 200 kg N/ha was added during the growing season as inorganic fertilizer. The maize cultivar used in the study has a growing cycle of ~120 days, and is amongst the most common type of cultivar used for this region. We also tested a second cultivar, planted 10 days earlier (day 110 vs. 120), starting in 2035 to simulate a likely adaptation strategy farmers are likely to adopt as climate continues to change. Two tillage practices were compared in this study, conventional and no tillage. The Conventional Tillage (CT) system included moldboard plowing at 25 cm before planting, with all the previous crop residues incorporated in the soil at the time of tillage. The No Tillage (NT) treatment assumed direct drilling of seeds, with all the previous crop residues retained on the soil surface. Rainfed and irrigation treatments were also compared. The irrigation treatment was set to automatically supply enough water to provide 100% of the evapotranspiration demand during the growing season.

### Climate Inputs

We applied modified change factor statistical downscaling analysis [34] based on an ensemble of the CMIP-5 GCMs [35] combined with historical weather observations from the NLDAS-2 (North American Land Data Assimilation System) forcing dataset [36]. This method preserves the month-to-month and year-to-year variability present in the historical record, while allowing both means and variance to be shifted by model forecasts. Traditional change factor (CF) downscaling uses the difference between averages of forecast and historical weather variables over some comparable time period, typically 30 years, to create projections of weather under a discretely altered climate. Static change factors are calculated monthly, and applied to finer scale weather data to create fixed-length scenarios. Here, we modify the CF method to allow variable monthly change factors through time to create continuous scenarios spanning the historical record through the end of the 21st century. Furthermore, we use ensemble GCM simulations of historical climate to remove observed trends prior to downscaling.

The first step in constructing the 121 year climate scenario was to build a historical observation dataset. Historical weather observations were based on the NLDAS-2 forcing dataset [37], which uses temporally and spatially downscaled air temperature and solar radiation from the North American Regional Reanalysis (NARR), and precipitation from gauge stations and NEXRAD radar. For the historical period (1979–2012), hourly NLDAS-2 values of precipitation, solar radiation, and temperature were aggregated to daily precipitation, solar radiation, as well as maximum and minimum temperature.

Next, monthly time-varying change factors for every variable were calculated using an ensemble generated from the historical and RCP 6.0 scenarios using a single run of all models in the Coupled Model Intercomparison Project-5 (CMIP-5) database [35, 36]. Monthly outputs from each model were first downscaled to a common 0.25° grid using bilinear resampling. Then, monthly averages across all models were calculated for each grid cell. Monthly averages from the neighboring four grid cells were then bilinearly interpolated to the study site location. A LOESS filter (calculated in R 3.0.2 with span parameter of 0.85) was used to smooth the time series of ensemble averages of monthly GCM outputs at the interpolated station location. The LOESS-filtered curve was then adjusted by subtracting out the bias between monthly averages of observations and the historical CMIP-5 scenario ensemble. Finally, each monthly trend was normalized using the mean of the historical period by subtracting the mean for daily maximum and minimum temperature, or dividing by the mean for precipitation and solar radiation.

After creating the change factors for each variable, these were used to detrended the 34-year period of historical observations, which were then replicated to create a complete continuous de-trended 121 year weather scenario. For each variable, the monthly CFs for each year were then subtracted from daily-aggregated historical observations (for maximum and minimum daily temperatures), or the observations were divided by the CF (for solar radiation and precipitation). The output of this detrending is a daily weather series that retains inter- and intra-annual variability due to broader climatic cycles, but without the secular trend due to global climate change. This detrended dataset was then replicated four times to create an adequate length scenario, which was then trimmed to the 1979–2099 period.

**Fig 1:**
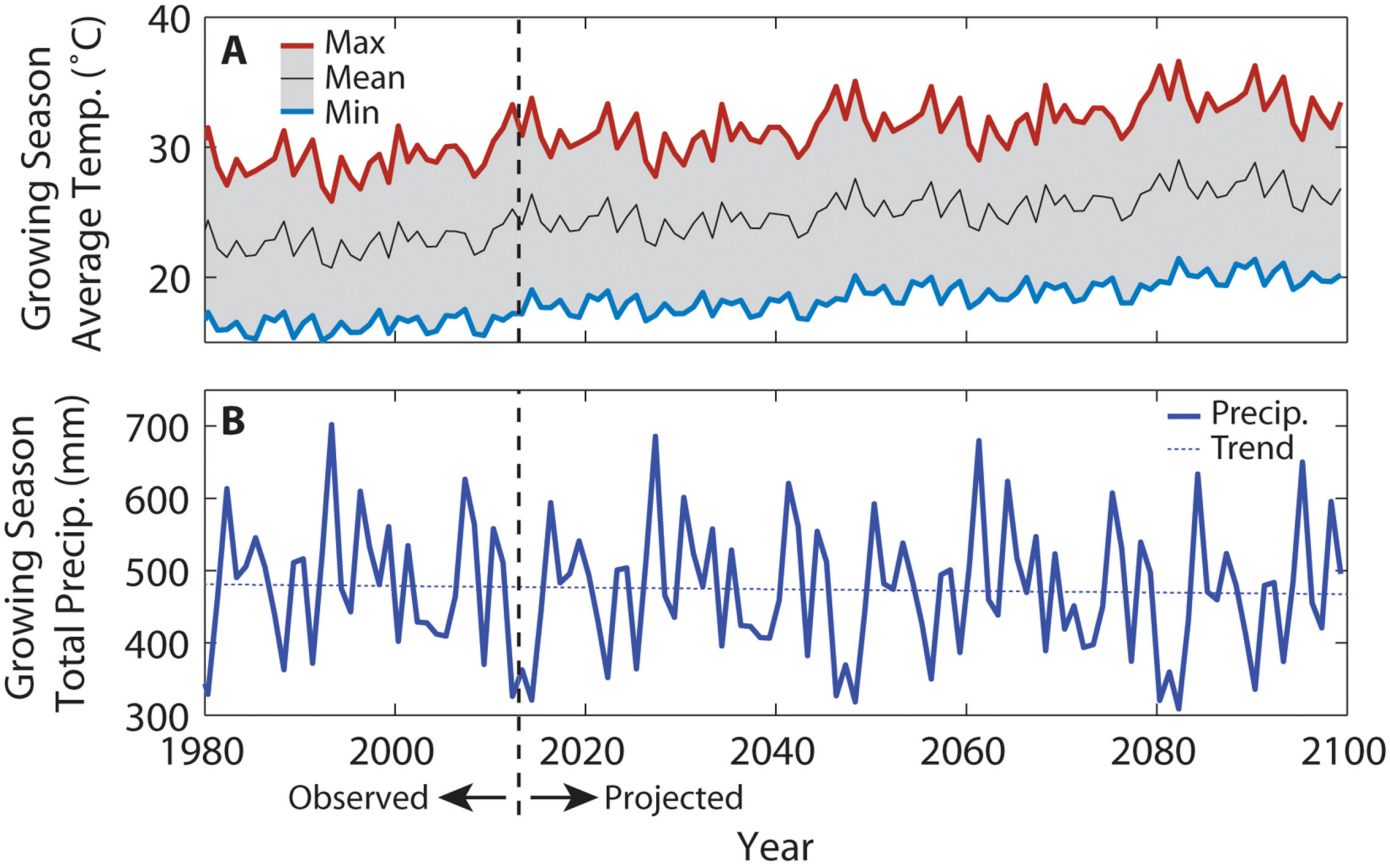
Growing season climate of the study site from 1980 to 2099. **A.** Average growing season (between planting and harvest) daily maximum, mean, and minimum temperatures, and **B.** total growing season precipitation. The average linear change in maximum and minimum temperatures across the 2013 to 2100 period, were 3.8°C/100y and 3.3°C/100y respectively. There is no significant trend in the growing season precipitation for this location.

The time-varying monthly change factors were applied to the 121 year detrended weather scenario. For maximum and minimum daily temperatures, the CFs were added to the detrended weather. CFs were then multiplied by precipitation and solar radiation to preserve days with zero observed flux. Annual averages of growing season temperatures and precipitation are presented in Fig 1.

The CO_2_ inputs to the model were taken from observations for the historical period and from the CMIP5 RCP 6.0 scenario for the period from 2013 to 2100. For this scenario, CO_2_ increases each decade from 325 in 1980, to 389 in 2010, and then to 669 in the projection to the end of the century.

## Results and Discussion

**Fig 2:**
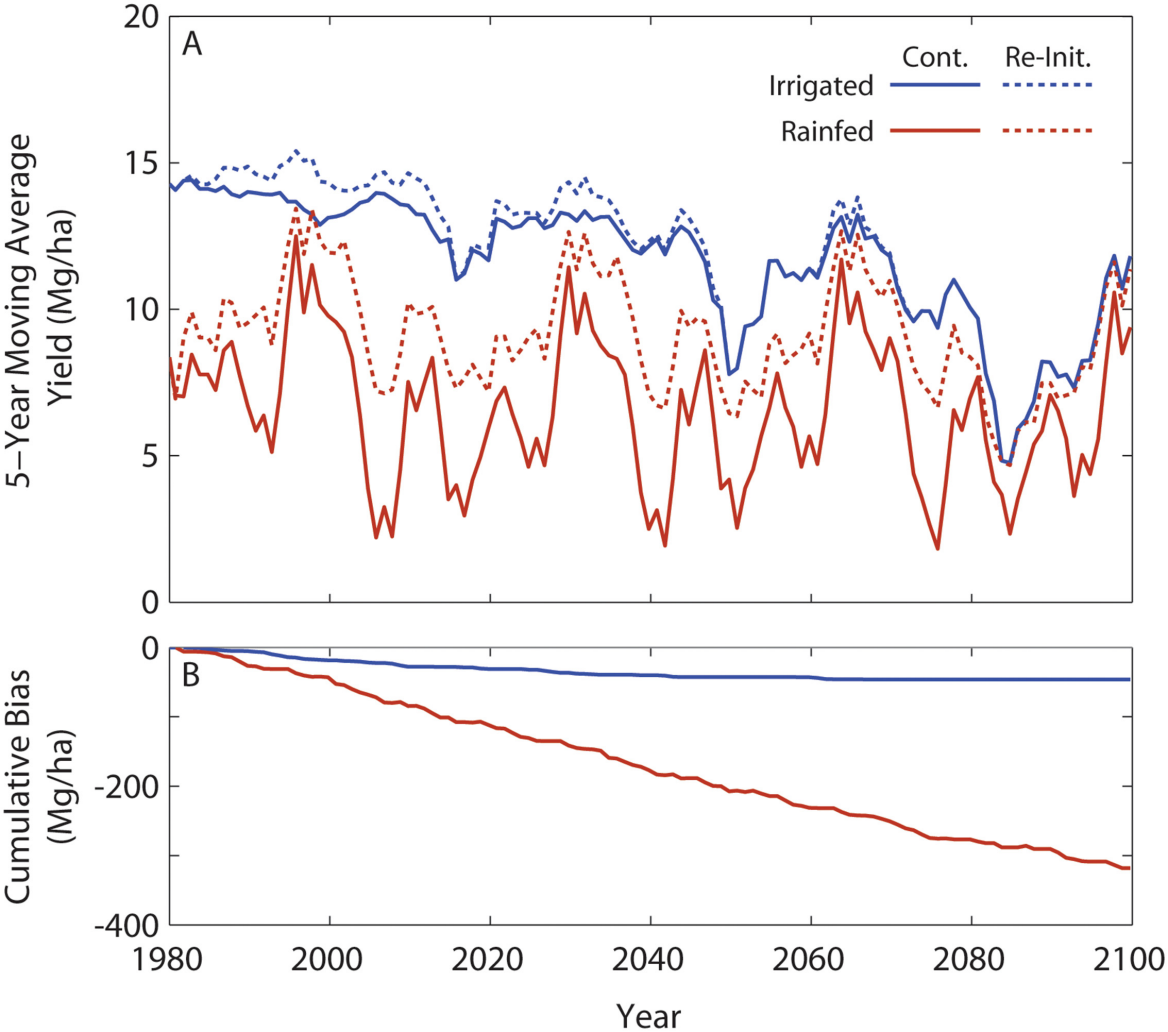
Comparisons of yield between continuous and re-initialized runs for both irrigated and rainfed treatments. **A.** Simulated rainfed and irrigated maize yields under projected climate with a fixed cultivar under no-till management. Continuous simulations (solid lines) vs. re-initialized on January 1 of each year (dashed lines) are shown for the NHP site. **B.** Cumulative bias in yield (continuous minus re-initialized).

Simulated corn yields for the 121 year continuous and re-initialized no-till scenario (Fig 2) showed similar patterns over time for the rainfed and irrigated treatments, but the yield difference between the continuous and re-initialized scenarios was much larger for the rainfed case. Simulated irrigated yields were slightly lower when the model was run in the continuous mode relative to yields in the re-initialized model. From 2050 onward, yields declined towards ~10 Mg/ha due to increasing temperatures, inhibiting grain filling, despite slightly higher productivity from CO_2_ enrichment with a current cultivar.

From 2003 to 2012, the predicted no-till maize yields were 12.9 Mg/ha and 5.3 Mg/ha for irrigated and rainfed treatments respectively. Over this decade, ~24% of maize acreage was irrigated in this county, resulting in an area weighted predicted yield of 7.14 Mg/ha. This compares favorably with the ten year average reported yield for all farms across Cuming county Nebraska of 7.96 Mg/ha (20–21), resulting in only ~10% error using an uncalibrated SALUS model. In addition, simulated irrigated yields ranged from ~13 to ~15 Mg/ha for the first 50 years, which is a typical yield for irrigated Nebraska maize [38].

Yield differences between continuous and re-initialized model runs for the rainfed scenario were substantially greater than those in the irrigated scenario (Fig 2A). The rainfed maize yields when the model was run in the continuous mode were highly variable with values ranging from a maximum of ~13 Mg/ha to minimum of ~2 Mg/ha. The rainfed maize yield in the re-initialized mode had lower fluctuations than the continuous simulation, with similar high values (~13 Mg/ha) but different lower values (~5 Mg/ha). These relative year-to-year deviations result in large cumulative yield differences (Fig 2B) that demonstrate re-initialization bias to be significant and (in this instance) positive for both irrigated and rainfed simulations.

The biases in simulated yields resulting from using re-initialized yields are summarized in Table 1 for four 30-year periods.

**Table 1:** Summaries of yield bias (continuous minus re-initialized) for irrigated and rainfed (Rain) treatments across four 30-year periods.

	Mean bias (Mg/ha)		Std. dev. bias (Mg/ha)		K-S p-value	
*Period*	*irrigated*	*rainfed*	*irrigated*	*rainfed*	*irrigated*	*rainfed*
*1980–2009*	-927.4	-2814.4	790.3	2848.0	0.0017	0.026
*2010–2039*	-413.0	-3081.9	565.5	2861.1	0.055	0.011
*2040–2069*	-201.5	-2454.8	462.5	2759.2	1.0	0.026
*2070–2099*	0.4	-2253.0	2.2	2610.4	1.0	0.011

Mean and standard deviations (Mg/ha) were calculated for the differences in annual yields for each period. Also, the p-value for a 2-sample Kolmogorov-Smirnov test is reported, testing the null hypothesis that the distribution in annual yields of the continuous and re-initialized yields are the same.

As depicted in Fig 2, the difference between continuous and re-initialized yields are substantially greater for the rainfed versus the irrigated treatment for all years. Additionally, the standard deviations are much larger.

**Fig 3:**
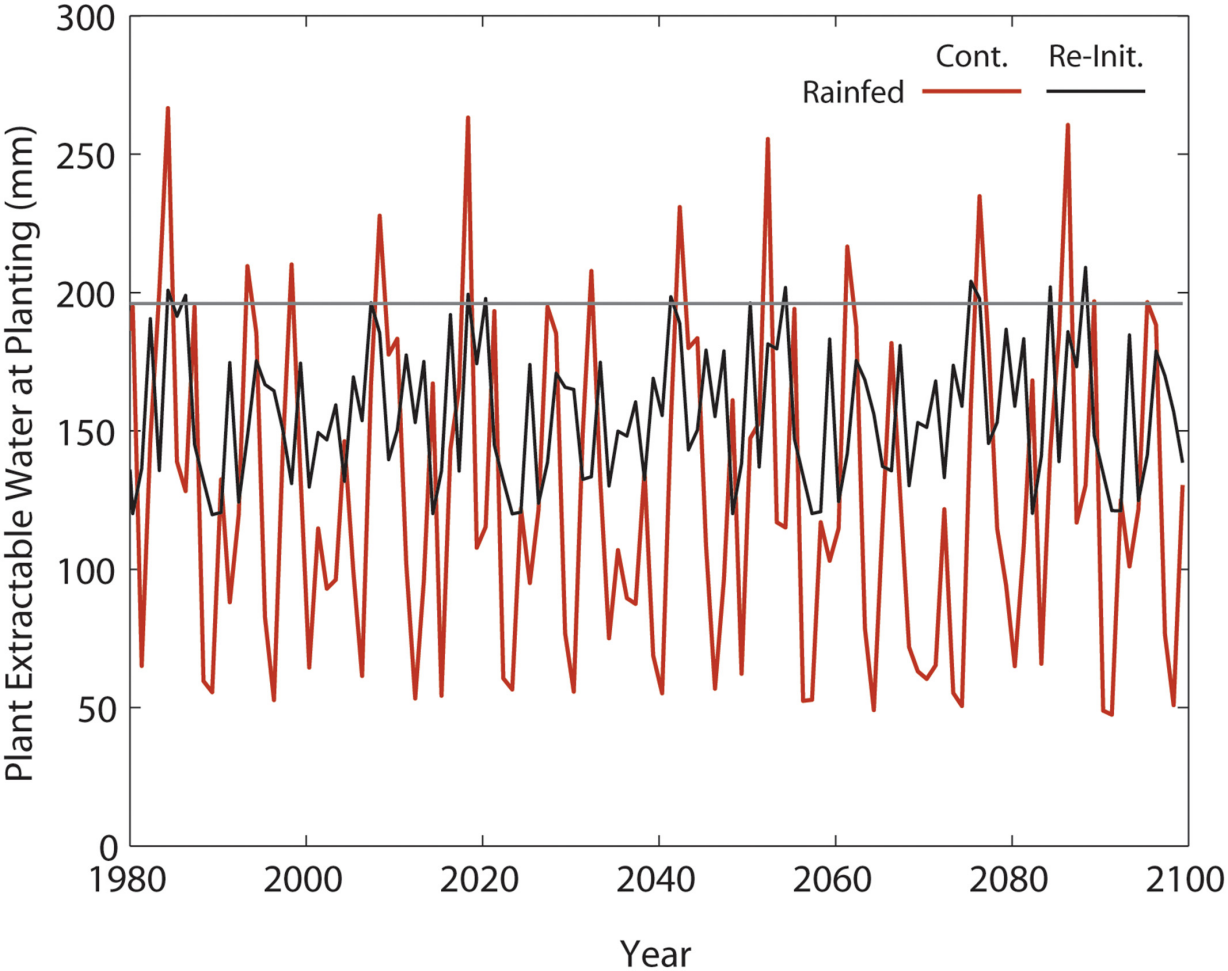
Predicted amount of Plant Extractable Soil Water (PESW) on the planting date (DOY 120) for rainfed maize. Red line shows the PESW for the continuous simulation; black line shows PESW re-initialized on January 1; straight grey line shows initial PESW for the re-initialized model. The soil profile was 150 cm thick.

A 2-sample Kolmogorov-Smirnov test was performed on the annual yield distributions for each paired simulation set (continuous vs. re-initialized) with the null hypothesis that the two distributions were equal. This test showed that for all years, the rainfed distributions are significantly different (p<0.05), while the irrigated distributions are significantly different only in the 1980–2009 period (p<0.01). Because soil water content differences are smaller between the simulation types in the irrigated treatment, the remaining bias is likely due to soil C and N. As temperatures warm in these scenarios, yields become limited by higher summer temperatures, rather than by soil C and N dynamics. The amount of PESW greatly varied over time in the continuous simulations in which soil water content is allowed to vary as a function of the prior year’s precipitation, runoff, recharge and crop water use. Values ranged from as low as 50 mm in some years up to 270 mm in others, in the continuous simulations, relative to ranges of 120 mm to 210 in the simulations re-initialized on January 1. If simulations fail to account for the soil conditions at planting time as impacted by fallow period weather, as the reinitialization does, there would unrealistically be more or less water and nutrients available for the crops at the beginning of each growing season, which will lead to biased results (Fig 3).

In this study, there was also less available N in the continuous simulation due to nitrate leaching (not shown), which occurs mostly during the fallow season [31]. This effect is not represented in re-initialized simulations, where high nitrate levels are always (and unrealistically) available each spring regardless of overwinter leaching rates. Nitrogen leaching is ignored upon re-initialization because most leaching occurs in the offseason, thus in the re-initialization the leached nitrogen is inappropriately added back to the soil profile via initial conditions.

**Fig 4:**
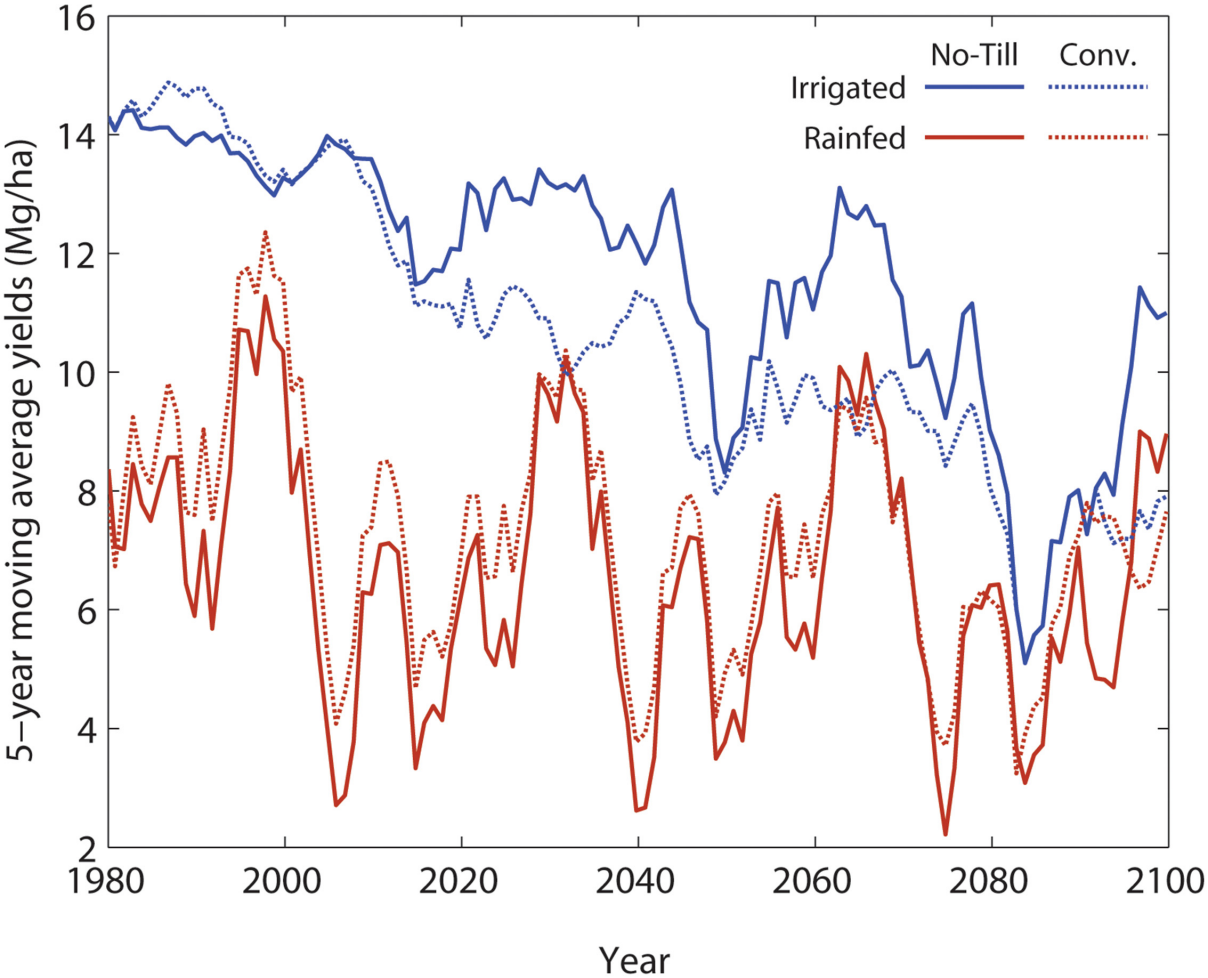
Plot of yields from no-till and conventional tillage for both irrigated and rainfed maize from 1980 to 2099 with continuous simulations.

Irrigated simulations clearly showed much higher yields (from ~6 to ~15 Mg/ha) for both no-till and conventional tillage compared to rainfed simulations (Fig 4).

Among tillage systems, conventional tillage initially produces higher yields than no-till. The no-till case showed higher yields during most years compared to conventional tillage after approximately 20 years of soil C accumulation,. Irrigated yields fluctuated over time with a declining trend of ~ 28% over the 121 year period. In the rainfed treatment, yields for both tillage practices showed large variability from ~2 to ~12 Mg/ha, with minor differences among the tillage systems. The rate of decline in rainfed simulations for both tillage systems was slightly lower.

**Fig 5:**
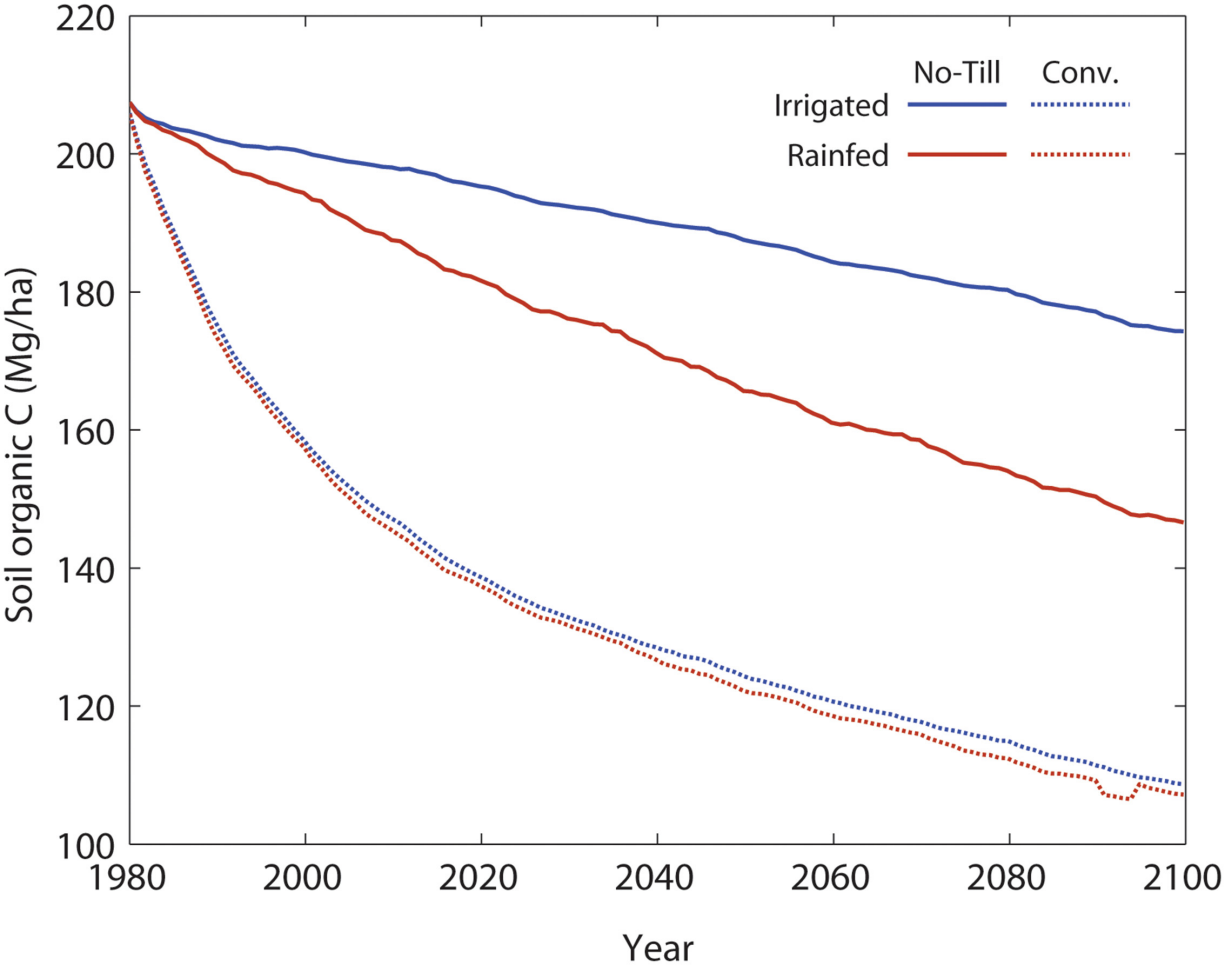
Projected soil carbon levels from 1980 through 2099 for treatments with rainfed and irrigated maize, and conventional and no-till agriculture.

Total soil organic carbon (SOC), depicted in Fig 5, declines through time for all the treatments. Conventional tillage has a sharper decline in SOC, resulting in long term lower yields.

Declines in yields for all treatments are caused by increased temperatures, which shorten the crop cycle. In turn, this results in lower residues being retained on the soil, which affects the amount of C in soils, pool sizes, and decomposition rates through time [34]. Irrigated no-till SOC decreased by ~15% over the 121 year scenario, while no-till rainfed SOC decreased by ~29%. Both conventional irrigated and rainfed scenarios experienced declines in total C by ~48% from original values by the end of the century.

**Fig 6:**
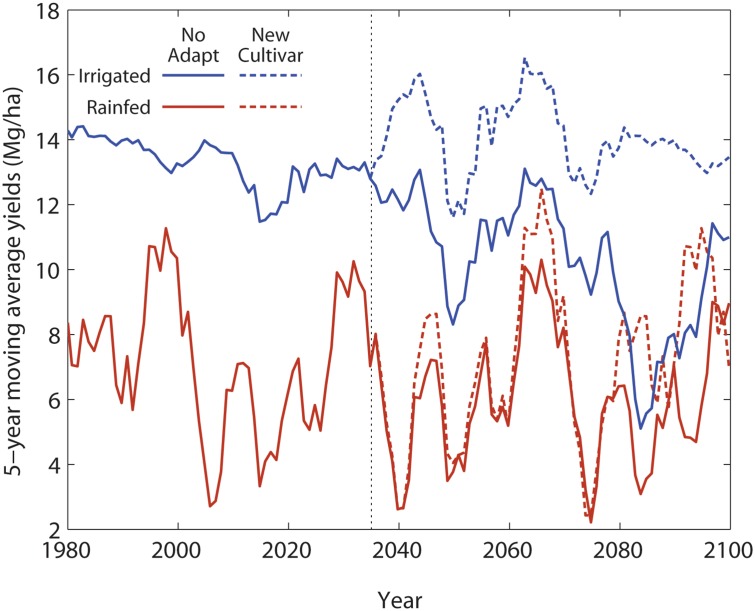
Plot of simulated maize yields with a potential adaptation strategy (dashed lines) of switching to a new cultivar and planting 10 days earlier in the year 2035 (vertical dashed line) to account for projected climate changes. These results are for both irrigated and rainfed no-till management from 1980 to 2099. Current management and cultivar are shown with solid lines.

Clearly farmers will adapt to climate change, rather than passively experiencing the extent of simulated declines. The most likely adaptation strategy for farmers will be to change planting dates and select cultivars that will benefit from the increased growing season length due to early planting and a longer frost free period [39]. Proper selection of adaptation strategies requires simulations that account for changes in soils as a result of different management strategies and cultivars. Fig 6 demonstrates that such adaptation strategies can successfully maintain simulated yields near levels experienced with current cultivars and planting strategies.

In particular, the results of the simulation with earlier planting (by 10 days) and adopting a modern cultivar with higher planting density and higher kernel setting efficiency (*New Cultivar* in Fig 6) demonstrated that yields can be maintained despite the negative impacts of projected temperature increases on the critical window of kernel setting.

## Conclusions

Because appropriate soil management can enhance the carryover of carbon and water, simulations that annually re-initialize pre-season soil carbon and water contents introduce a yield bias that obscures the potential for soil management to ameliorate the deleterious effects of rising temperatures and greater rainfall variability. This bias is particularly strong for rainfed cropping systems in relatively low-carbon soils. By removing this bias it becomes possible to express the ability of different soil and crop management strategies to mitigate otherwise substantial challenges to food security. Rainfed cropping systems will benefit from additional soil carbon buildup, which improves overall soil biophysical and chemical properties. Only by using models that avoid re-initialization and thus accurately predict beginning-of-season conditions based on recent and long-term soil environmental change can we appropriately evaluate potential adaptation strategies for agriculture, such as earlier planting, new cultivars, and better soil management.
